# Application of oral nutritional supplements to control body weight loss in postoperative patients suffering from solid tumors: a systematic review and meta-analysis

**DOI:** 10.3389/fnut.2025.1476463

**Published:** 2025-02-12

**Authors:** Ying Liu, Zhen Wu, Tingting Shao, Wanzhen Zheng, Jing Huang

**Affiliations:** Department of Integrated Traditional Chinese & Western Medicine Oncology, Hangzhou Cancer Hospital, Hangzhou, China

**Keywords:** dietary supplements, body weight, neoplasms, surgery, meta-analysis

## Abstract

**Objective:**

This study aims to summarize the impact of oral nutritional supplements (ONSs) on mitigating body weight loss (BWL) in patients following surgical treatment for solid tumors.

**Methods:**

A systematic and comprehensive search of four major publicly available databases was conducted up to May 2024 to identify studies for inclusion in the analysis. Data from eligible studies were extracted, and pooled mean differences (MD) along with their 95% confidence intervals (CIs) for BWL were computed.

**Results:**

A total of 12 randomized controlled trials (RCTs) with 2,268 participants were finally included. The group receiving oral nutritional supplements demonstrated a statistically significant reduction in weight loss compared to the control group, with a mean difference of 1.11 (95% CI: 0.52–1.70), an *I*^2^ statistic of 97.0%, and a *p*-value less than 0.01.

**Conclusion:**

The meta-analysis provide evidence that ONSs effectively reduce BWL in postoperative patients with solid tumors. Additionally, ONS with lower daily caloric intake demonstrated superior efficacy in reducing BWL.

## Graphical abstract

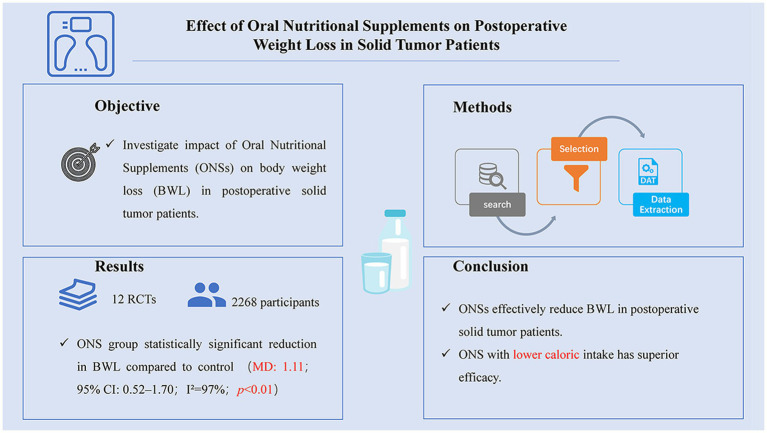

## 1 Introduction

Cancer continues to pose a significant public health challenge in the 21st century, accounting for approximately one in six deaths globally (16.8%). In 2022, it is estimated that there will be 20 million new cancer cases and 10 million cancer deaths worldwide (1). Despite advances in systemic treatments, including targeted therapy and immunotherapy, surgical resection remains a cornerstone in the management of solid tumors (2, 3). In recent years, with advancements in surgical techniques (such as laparoscopy, robotic surgery, and reconstructive procedures) and changes in treatment philosophy, surgical interventions in cancer treatment have increasingly focused not only on achieving radical cure but also on preserving form, function, and quality of life (4, 5). Despite these advancements, postoperative complications, particularly malnutrition and weight loss, remain critical issues that need addressing.

Malnutrition and weight loss are prevalent among cancer patients, with 20 to 40% experiencing body weight loss (BWL) at diagnosis (6, 7). The physiological stress and metabolic changes induced by surgical intervention can exacerbate pre-existing nutritional deficiencies, leading to further body weight loss (BWL) (8, 9). Weight loss was associated with an increased risk of postoperative complications, reduced chemotherapy tolerance, a marked decline in performance status and quality of life, and decreased survival rates (10–12). Therefore, nutritional intervention and the management of weight loss are essential.

Oral nutritional supplements (ONSs) play a pivotal role in addressing these challenges. ONSs are multi-nutrient products available in liquid, semisolid, or powder forms that deliver both macro-and micronutrients. They are intended to boost nutritional intake in patients whose dietary needs cannot be met through regular food alone. Unlike vitamin and mineral supplements in pill form, ONSs offer comprehensive nutritional support, making them indispensable in managing malnutrition and preventing weight loss across diverse medical conditions (13). In the context of cancer care, ONSs are extensively used across various cancer types, stages, and treatments, including chemotherapy, radiotherapy, and surgery (14, 15). They are widely recognized as one of the most critical forms of nutritional support for cancer patients and those recovering from surgery (16).

However, the effectiveness of ONSs in reducing body weight loss (BWL) among cancer patients undergoing surgery has yielded conflicting results (17–20). Additionally, there is a notable lack of rigorous analytical studies examining the potential covariates that may influence these outcomes.

This meta-analysis aimed to assess the effect of ONSs on BWL in postoperative patients with solid tumors and to further explore the potential moderating variables as influencing factors. By doing so, it seeks to provide a clearer understanding of the role of ONSs in enhancing postoperative recovery and overall patient well-being.

## 2 Materials and methods

### 2.1 Statement

This study was performed in adherence to the Preferred Reporting Items for Systematic Reviews and Meta-Analyses (PRISMA) guidelines, ensuring rigorous and transparent reporting of systematic review and meta-analysis methodologies (21). The study utilized publicly available data from databases including PubMed, Embase database, Web of Science and Cochrane databases, and did not involve human participants. Thus, it did not require submission for institutional review board approval or informed consent.

### 2.2 Search strategy

A thorough search was performed covering literature from inception through May 2024. The detailed search strategy incorporated a combination of the MeSH term “Dietary Supplements” OR the keywords “Dietary Supplement*,” “Food Supplement*,” “Oral nutritional supplements,” “Nutraceutical*”; MeSH term “Neoplasms” OR the keywords “Tumor*,” “Neoplasm*,” “Cancer*” and “Neoplasia*”; the keywords “Surgery,” “Operation,” “Resection” OR “Microsurgery”; MeSH term “Body Weight” OR the keywords “Body Weight*.” PubMed and Cochrane utilize the same MeSH for indexing. In Embase, however, searches are based on Emtree. If the terms do not match, we also expand to the closest related terms. This search is language-and study-type agnostic. This meta-analysis exclusively includes randomized controlled trials (RCTs). Two independent researchers manually searched additional references from the reference lists of pertinent studies as well as review articles to identify further references and reviewed all pertinent texts tables, and figures to extract data.

### 2.3 Study selection

Studies meeting the following inclusion criteria would be selected: (1) patients who have been diagnosed with malignant tumor and have subsequently undergone surgical treatment; (2) interventions including ONSs; (3) Comparisons were made based on usual, standard or regular diet categories; (4) outcomes assessed as mean differences in body weight; and (5) RCTs. Duplicate publications, animal studies, and articles without original data (including reviews, abstracts only, letters, comments, editorials, meetings, and case reports) were excluded. Studies without comparison groups or complete data were also excluded.

### 2.4 Data extraction

Two researchers separately assessed the titles, abstracts, and complete documents of the articles based on pre-defined inclusion criteria. To ensure a thorough and objective evaluation of the data, any discrepancies or disagreements between the reviewers were resolved through a process of discussion and consensus-building. When agreement could not be achieved, a collaborative review involving all researchers was carried out to determine article inclusion. Data extraction was carried out using specialized forms designed for this purpose. To ensure the meta-analysis’s accuracy and completeness, comprehensive checks were performed on the references from each included study to identify and remove any potential overlaps.

Extracted parameters from the trials included the first author, year of publication, study country, study design, type of ONS nutrients, controls conditions, intervention duration, characteristics of the study population, such as patients count, age distribution, female ratio, disease type, daily kilocalorie intake, TNM stage and body weight data. For studies lacking standard deviation reports, pooled standard deviations were estimated for each group.

### 2.5 Statistical analysis

Mean differences (MD) along with their associated 95% confidence intervals (CIs) were computed to assess the continuous variable of body weight loss (BWL). To obtain the pooled overall MD and 95% CIs for the outcomes, a random-effects model was utilized, analyzed with restricted maximum likelihood (REML) estimation (22). The choice between a fixed-effects model and a random-effects model was based on the level of heterogeneity assessed. Heterogeneity among included studies was valuated using Cochrane’s Q test and the Higgins’ *I*^2^ values. An *I*^2^ value of less than 50% was interpreted as low heterogeneity, indicating that a fixed-effects model was appropriate. Conversely, an *I*^2^ value greater than 50% suggested substantial heterogeneity, warranting the use of a random-effects model. Statistical significance of heterogeneity was determined with Cochran’s Q test, where a *p*-value below 0.05 was considered indicative of significant heterogeneity (23).

To investigate factors moderating BWL, we conducted meta-regression analyses on continuous variables including patient number, age, percentage of females and intervention duration. For categorical variables such as daily calorie intake, type of disease, and country, we performed meta-analysis of variance. REML estimation was employed to assess variance in true effects and a examine potential moderating factors. *p*-values of 0.05 or less, or 95% CI that did not include zero (mean difference = 0), were deemed statistically significant. In this meta-analysis, R software (R Foundation for Statistical Computing; version 4.4.1) was utilized for data processing and the results were presented in the form of figures.

### 2.6 Publication bias assessment and quality assessment

Publication bias was evaluated through the use of a funnel plot, with a symmetrical distribution of studies within the plot suggests indicating a lack of bias. To more precisely quantify the extent of publication bias, we employed Egger’s test as an additional analytical method (23–25).

The quality of included RCTs was evaluated using the Cochrane Collaboration’s Risk-of-Bias (RoB) 2.0 tool. This comprehensive assessment tool evaluates potential bias across five key domains. Each domain was systematically rated to determine the risk of bias as high, low, or some concerns. In the RoB 2.0 item, an overall risk rating of “high” is assigned if any one of the five domains is rated as “high,” or if two or more domains are rated as “some concerns” (26).

## 3 Results

### 3.1 Study selection and characteristics

**Figure 1:**
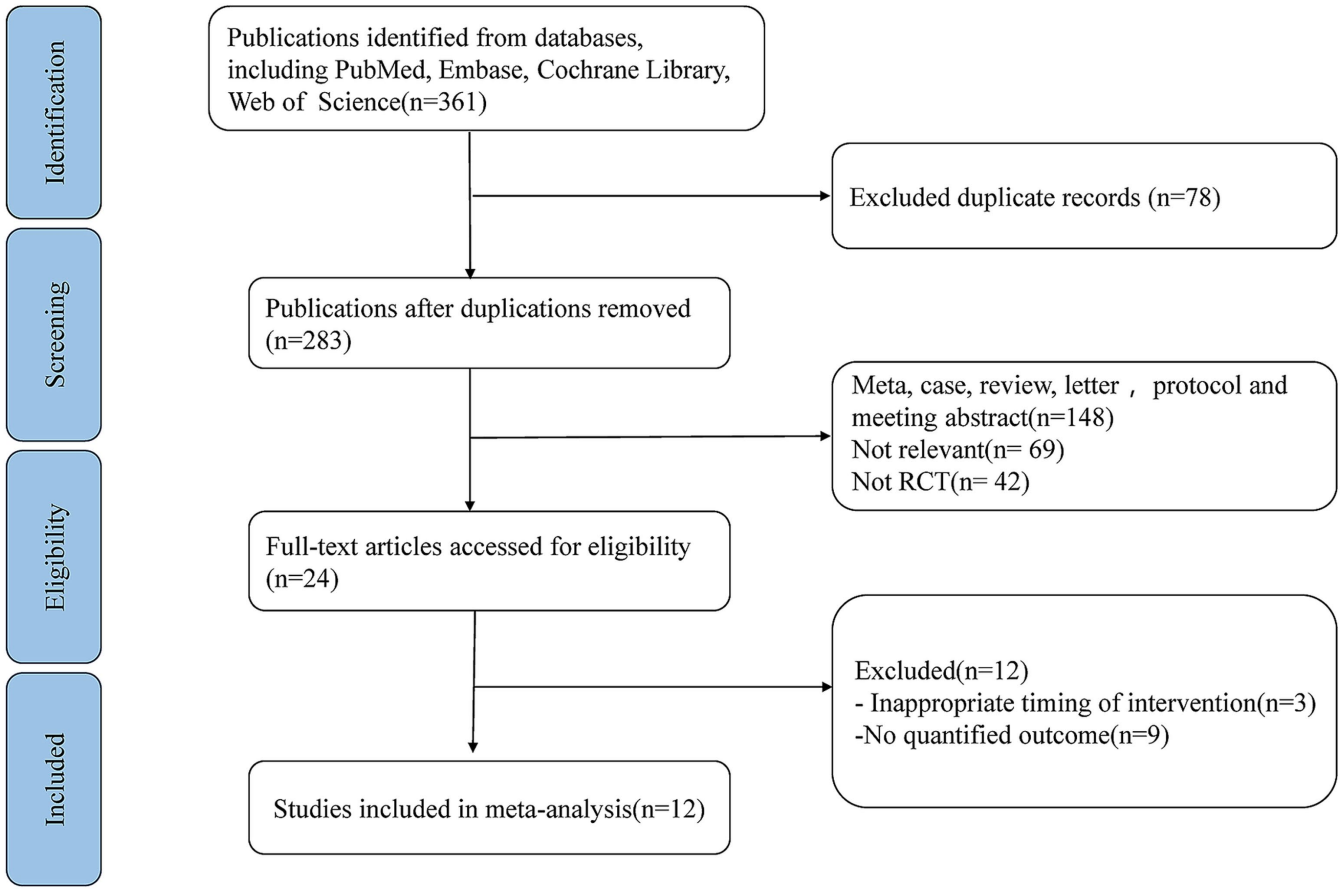
PRISMA flow diagram illustrating the study selection process for systematic review and meta-analysis.

The initial search identified 361 publications across PubMed, Web of Science, Embase databases and the Cochrane Library. Among them, 78 articles were excluded due to duplication. After reviewing titles as well as abstracts, 259 articles were also eliminated for various reasons. The remaining 24 full-text studies were subjected to a comprehensive and detailed review. 12 articles were further removed, including 9 articles due to lack of quantitative outcomes and 3 articles due to inappropriate timing of intervention. Ultimately, twelve studies fulfilled the selection criteria and were incorporated into this meta-analysis (27–38) (Figure 1). These 12 cohort studies encompassed a total of 2,268 cancer patients diagnosed with stomach, colorectal, head and neck, or bladder cancer. The studies were conducted in Asia and the Americas, specifically with five from Japan, four from China, one from Thailand, one from Brazil, and one from the United States. The key characteristics of these included RCTs were summarized in Table 1.

**Table 1:** Characteristics of the included studies.

Study (year)	Country	Study design	Disease type	Average age (Years)	No. of patients (% Female)	Consumption (Kcal/Day)	ONSs nutrient	Controls	Intervention duration (Months)	TNM stage (%)
Miyazaki 2021 (31)	Japan	RCT	Gastric cancer	66.65	880 (35.5)	400 (310)	ONSs	Regular diet	3	I (62.8), II (19.0), III (15.9), IV (2.3)
Meng 2021 (32)	China	RCT	Gastric cancer	59.93	337 (32.3)	500 (370)	ONSs	Dietary advice alone	3	I (25.8), II (28.8), III (38.6), IV (6.8)
Tan 2021 (29)	China	RCT	Colorectal cancer	59.15	212 (35.38)	500 (410)	ONSs	Dietary advice alone	3	I (14.6), II (40.1), III (40.1), IV (5.2)
Jantharapattana 2020 (33)	Thailand	RCT	head and neck cancer	57.35	62 (19.35)	630	ONS plus EPA (2.2 g/d)	Standard formula	1	NR
Yang 2020 (28)	China	RCT	Colorectal cancer	58.64	85 (38.8)	500	ONSs	Dietary advice alone	3	NR
Ritch 2019 (30)	the United States	RCT	Bladder cancer	68	52 (11.5)	700	ONSs	Multivitamin	2	NR
Aoyama 2019 (38)	Japan	RCT	Gastric cancer	65.34	123 (27.6)	600	ONS plus EPA (2.2 g/d)	Standard diet	1	NR
Zhu 2019 (27)	China	RCT	Gastric cancer, Colorectal cancer	59.5	114 (31.58)	500	ONSs	Dietary advice alone	3	NR
Feijó 2019 (37)	Brazil	RCT	Gastric cancer	58	68 (35.3)	600	ONS plus EPA (3.2 g/d)	Standard formula	1	I (4.4), II (25), III (45.6), IV (7.4)
Ida 2017 (35)	Japan	RCT	Gastric cancer	65.34	123 (27.6)	600	ONS plus EPA (2.2 g/d)	Standard diet	1	I (39.8), II (31.7), III (28.5), IV (0)
Hatao 2017 (36)	Japan, Taiwan	RCT	Gastric cancer	64.8	113 (38.9)	400	ONSs	Usual postoperative diet	3	I (53.1), II (22.1), III (24.8), IV (0)
Imamura 2016 (34)	Japan	RCT	Gastric cancer	66.17	99 (29.7)	300	ONSs	Regular diet	2	I (60.4), II (21.6), III (17.1), IV (0.9)

NR, not reported; ONSs, oral nutritional supplements; Kcal: kilocalorie; EPA, eicosapentaenoic acid; No, number.

### 3.2 Outcomes

**Figure 2:**
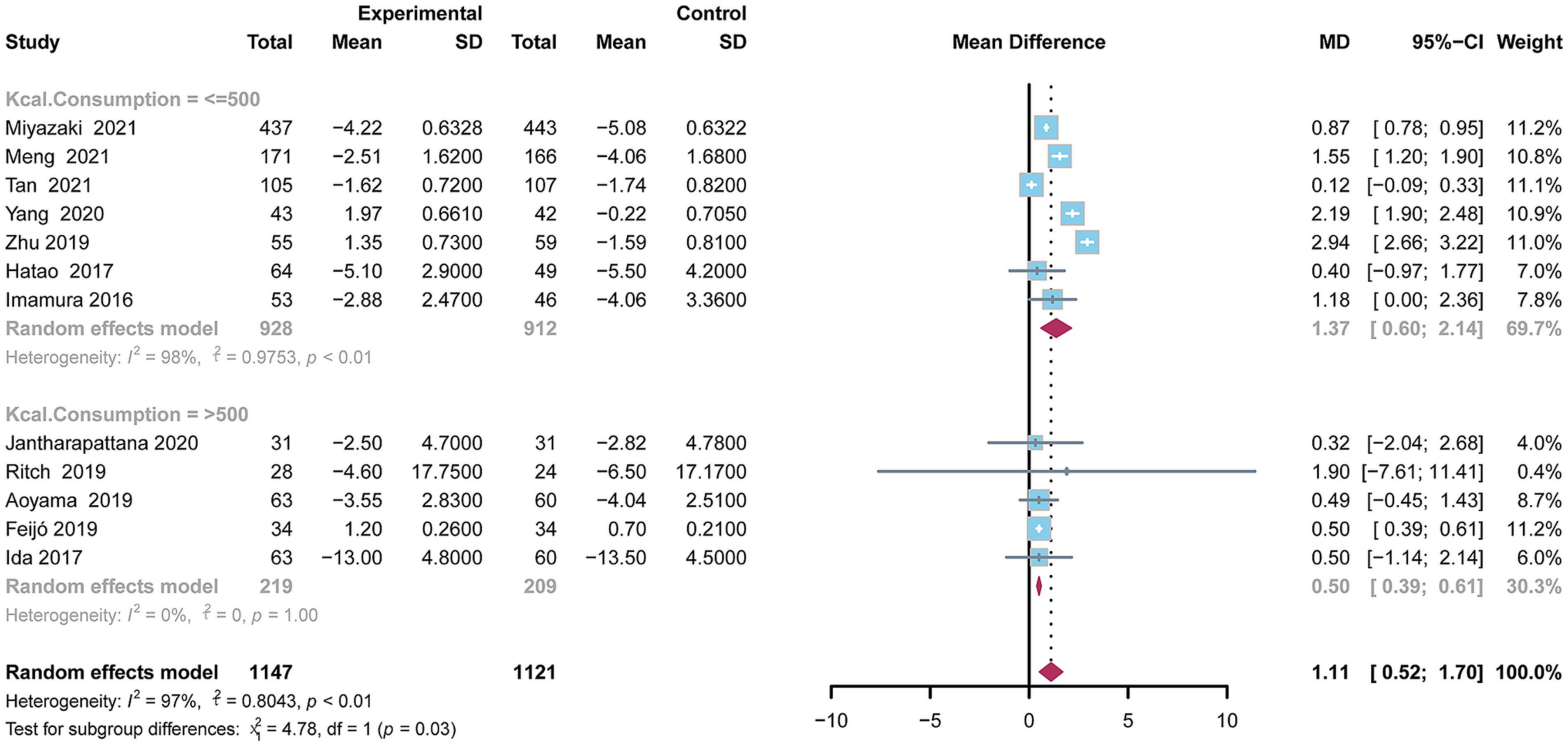
Forest plot depicting the difference in body weight between the oral nutritional supplements and control groups using a random-effects model.

This analysis demonstrated a statistically significant reduction in BWL in the oral nutritional supplement (ONS) group compared to the control group with a mean difference (MD) of 1.11 (95% CI: 0.52–1.70). Due to significant heterogeneity (*I*^2^ = 97.0%, *p* < 0.01), a random-effects model was applied for the pooled analysis of BWL. To further assess the impact of daily ONS consumption on BWL, a subgroup analysis was carried out. In the subgroup consuming less than 500 kilocalories per day, the pooled MD was 1.37 (95% CI: 0.60–2.14), indicating statistical significance (*p* < 0.01), though the Higgins’ *I*^2^ value was 98.0%, reflecting considerable heterogeneity. Conversely, in the subgroup consuming over 500 kilocalories per day, the pooled MD between the ONS group and control groups was 0.50 (95% CI: 0.39–0.61), also statistically significant (*p* = 0.01) with a Higgins’ *I*^2^ value of 0.0%, indicating low heterogeneity (Figure 2).

### 3.3 Moderator analysis

Meta-regression and meta-analysis of variance were conducted to investigate potential moderators influencing BWL, such as daily nutritional intake as well as the study country was conducted (Table 2). The analysis revealed significant heterogeneity in the two variables, daily consumption and study country. Patients in the ≤500 kcal group experienced greater BWL compared to those in the >500 kcal group (*p* = 0.0287). Additionally, there were statistically significant differences in BWL between countries (*p* < 0.0001), with China (MD = 1.70, 95% CI = 0.52–2.88) and the United States (MD = 1.90, 95% CI = −7.61–11.41) showing higher mean differences than Japan (MD = 0.86, 95% CI = 0.78–0.94), Thailand (MD = 0.32, 95% CI = −2.04–2.68), and Brazil (MD = 0.50, 95% CI = 0.39–0.61). Furthermore, differences were observed between disease types, with patients in the gastrointestinal tumor group showing better improvement in BWL (MD = 1.14, 95% CI = 0.52–1.75) compared to those in the non-gastrointestinal cancer groups (MD = 0.41, 95% CI = −1.88–2.70), although this result did not achieve statistically significant (*p* = 0.5494), probably due to insufficient sample size. Longer intervention duration was associated with reduced BWL (*β* = 0.46), though this finding also lacked statistical significance (*p* = 0.1512).

**Table 2:** Impact of modifying factors on the efficacy of oral nutritional supplements.

Variables	*k*	*β*	MD	95% CIL	95% CIH	*p*
No. of patients	12	−0.0003		−0.0028	0.0022	0.8005
Age	12	−0.0661		−0.2387	0.1065	0.4532
Female rate	12	0.0193		−0.1074	0.1459	0.7656
Duration (Month)	12	0.4561		−0.1667	1.0790	0.1512
Kcal consumption						0.0287
≤500	5		1.3687	0.5979	2.1394	
>500	7		0.4997	0.3885	0.6108	
Disease						0.5494
Gastrointestinal cancer	10		1.1358	0.5237	1.7479	
Non-gastrointestinal cancer	2		0.4117	−1.8787	2.7020	
Country						< 0.0001
Japan	5		0.8620	0.7793	0.9448	
China	4		1.6978	0.5198	2.8758	
Thailand	1		0.3200	−2.0398	2.6798	
US	1		1.9000	−7.6085	11.4085	
Brazil	1		0.5000	0.3877	0.6123	

k, number of effect sizes; β, regression coefficient; MD, mean difference; *p*-value from the meta-regression analysis using the restricted maximum likelihood. CIL, confidence interval low; CIH, confidence interval high.

### 3.4 Publication bias and sensitivity analysis

The assessment of publication bias associated with this study was presented using a funnel plot (Supplementary Figure S1), which visually indicated that the mean differences were symmetrically distributed. Egger’s test further supported the absence of publication bias (*p* = 0.4562).

The stability of the mean difference for body weight loss was tested using the trim and fill method under a random-effects model. After imputing two hypothetical studies, the adjusted pooled MD (MD = 0.70, 95% CI = 0.0034–1.39, *p* = 0.0489) was not significantly different from the original pooled MD, indicating that the initial conclusions remained robust (Supplementary Figure S2). Additionally, the pooled MD showed no substantial change regardless of which study was excluded in the sensitivity analysis (Supplementary Figure S3).

### 3.5 Quality assessment

**Figure 3:**
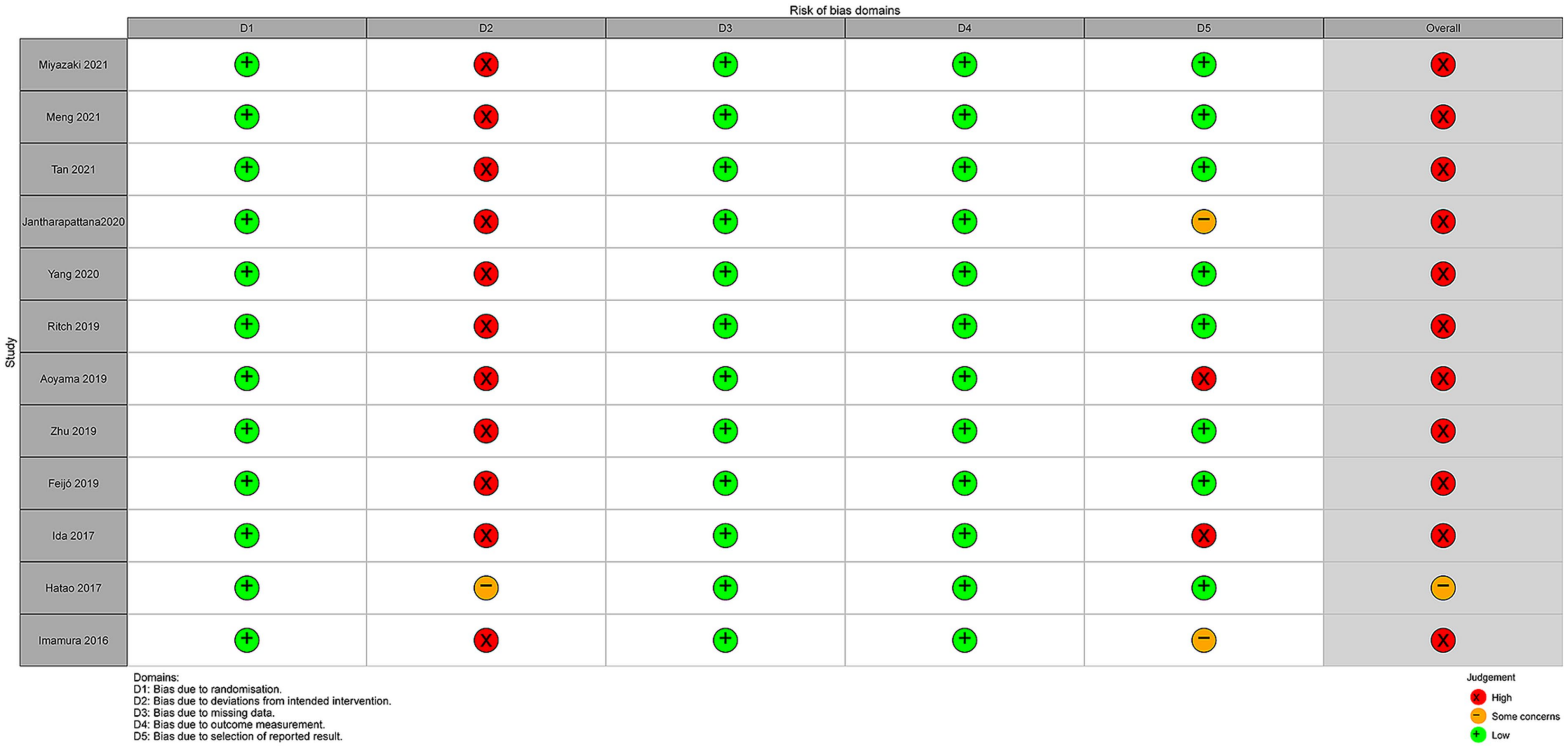
Risk of bias assessment using the Risk of Bias 2.0 tool for the 12 included studies.

Twelve studies were assessed for risk of bias using the five domains of the RoB 2.0 tool (Figure 3). The assessment results showed that the overall risk of bias of 11 studies was rated as “high,” except for 1 study which was rated as “some concerns.” This outcome is largely attributed to the RoB 2.0 criteria, where a study is classified as high risk if any domain is deemed high risk. Specifically, in the domain of bias related to deviations from intended interventions (D2), eleven studies were rated as “high risk,” while 1 study was rated as “some concerns,” primarily because most studies were unable to blind the oral nutritional supplement (ONS) intervention due to its nature. Additionally, in the area of bias related to selective reporting (D5), 2 studies assessed as having “high risk” and while another two were evaluated as “some concerns.”

## 4 Discussion

This meta-analysis represents the first study to demonstrates that oral nutritional supplements can improve body weight loss in patients after solid tumor surgery. Notably, ONS with lower caloric intake was found to be more effective compared to higher-calorie supplements.

Malnutrition, often manifesting as unintentional weight loss, is a prevalent and serious issue among cancer patients (39). This condition may result from reduced food intake, underlying catabolic processes, metabolic and inflammatory alterations, and the adverse effects of anticancer treatments (40). According to reports, Malnutrition is highly prevalent in cancer patients undergoing surgery, with a prevalence of 65% in gastrointestinal tumors (41), 60% in head and neck cancer (42) and 55% in urological tumors (43). Due to the association between BWL and postoperative chemotherapy tolerance and prognosis, it is crucial to adopt proactive nutritional intervention strategies to improve BWL. Nutritional intervention for cancer patients includes comprehensive nutritional assessment, optimization of caloric and protein intake, management of specific symptoms, enhancement of appetite, and the use of supportive nutritional supplementation (including oral nutritional supplements). The ESPEN practical guidelines recommend early enteral nutrition for those undergoing surgery, especially for upper gastrointestinal surgery (16).

Oral nutritional supplements (ONS) have shown broad application value as a comprehensive nutritional strategy in the management of various clinical conditions. For example, in patients with chronic obstructive pulmonary disease (COPD) (44), chronic kidney disease (45), geriatric frailty syndrome (46), those undergoing orthopedic surgery (47), and individuals in intensive care (48), ONS have been shown to improve nutritional status, reduce complications, support recovery, and enhance quality of life. ONS provides essential macronutrients and micronutrients that may be compromised in postoperative cancer patients due to reduced oral nutritional intake, increased metabolic demands, and altered gastrointestinal function. In addition, specific components of ONS, such as omega-3 fatty acids and specific amino acids, may have anti-inflammatory and immunomodulatory effects that helps with recovery after surgery and may contribute to better overall outcomes (49). In the past, meta-analyses on ONS to reduce BWL after surgery mostly focused on gastrointestinal tumors, with no consensus reached on the conclusions (17–20). Notably, there remains a significant gap in the literature, as no meta-analysis to date has specifically investigated the impact of ONS on postoperative weight loss in patients with solid tumors across a broader spectrum of cancer types. Addressing this gap, the present study provides the most up-to-date evidence, demonstrating that ONSs are effective in improving the postoperative BWL of patients with solid tumors. By focusing on this understudied population, our research aims to fill a critical void in the current understanding and to offer valuable insights that could inform clinical nutritional strategies for postoperative cancer patients.

The meta-analysis shows that ONS with a daily intake of less than 500Kcal has a better effect on improving body weight than the group with a higher intake. This finding suggests that a lower caloric threshold may be more efficient in promoting weight gain or maintenance in certain populations, possibly due to better compliance or reduced gastrointestinal discomfort associated with low calorie consumption. A study reported that a 600-kcal daily eicosapentaenoic acid-enriched oral nutritional supplement did not significantly reduce BWL compared to a standard diet following total gastrectomy for gastric cancer (35). The researchers speculated that this may be because patients were affected by the satiety caused by ONS, especially after gastrectomy, reducing normal diet intake. This may be due to functional changes caused by cancer surgery, resulting in decreased absorption, therefore, high calorie intake may be ineffective for BWL (50). In addition, ONS should be advised for consumption between or after meals, rather than before meals or as a substitute for them (13). This approach maximizes its effectiveness without diminishing appetite for regular meals.

In addition to caloric intake, several factors may influence the effectiveness of ONS in postoperative patients with solid tumors, including dietary habits, comorbidities, and concomitant medications. Postoperative pain, gastrointestinal dysfunction, and structural changes in the digestive tract often result in reduced food intake. Furthermore, unhealthy dietary preferences or imbalanced eating patterns can exacerbate this issue, increasing the risk of weight loss (51, 52). The presence of common comorbidities, such as diabetes and cardiovascular diseases, complicates the use of ONS in managing postoperative weight loss in cancer patients, as these comorbidities may reduce the potential benefits of ONS by disrupting metabolic pathways, promoting catabolism, and increasing susceptibility to infection (16, 53, 54). Additionally, certain medications, such as statins, may interfere with the efficacy of ONS by counteracting the anabolic effects of protein supplementation, particularly through their impact on lipid metabolism and muscle mass (55).

However, this meta-analysis has several limitations. First, the overall quality of the studies included was low due to a high risk of bias. Specifically, 11 of 12 studies were rated as high risk in the intervention deviations bias domain, which led to an overall assessment of high risk. According to the RoB 2.0 criteria, if any single domain is rated as high risk, the entire study is classified as high risk. For behavioral interventions like oral nutritional supplements, blinding comparisons can be particularly challenging. Despite this, we consider that the quality level of these RCTs is acceptable. Second, the variation in the type, dosage, and duration of oral nutritional supplement interventions among the 12 studies may have impacted the overall effect size. Third, the studies varied in cancer type and definitions of tumor stage, with a predominance of gastrointestinal cancers and limited representation of other cancer types, potentially affecting the overall results. Fourth, the control groups differed across studies, including regular diet, standard formula, dietary advice alone, and usual postoperative diet. Fifth, 11 of 12 included studies were conducted in Asia, with a significant number originating from Japan. This geographical concentration may restrict the applicability of the pooled findings to populations from other ethnic backgrounds. Further multicenter, high-quality RCTs with standardized protocols are required to confirm our findings and yield more conclusive recommendations.

## 5 Conclusion

This analysis provides new and compelling evidence to support the use of oral nutritional supplements to decrease body weight loss in postoperative patients with solid tumors. Incorporating ONS into routine postoperative care may improve nutritional outcomes and aid in the recovery process. Additional studies are necessary to investigate the effects of tailored nutritional interventions on body weight loss and overall prognosis in postoperative cancer patients.

## Data Availability

The original contributions presented in the study are included in the article/Supplementary material, further inquiries can be directed to the corresponding author.
