# Supplementary material for: Application of oral nutritional supplements to control body weight loss in postoperative patients suffering from solid tumors: a systematic review and meta-analysis
Source: Front Nutr. 2025 Feb 12;12:1476463. doi: 10.3389/fnut.2025.1476463 (PMC11860093; doi:10.3389/fnut.2025.1476463)
Supplement: Supplementary file 1 [file Image_1.pdf]

Application of oral nutritional supplements to control body weight loss in postoperative patients suffering from solid tumors: a systematic review and meta-analysis

Ying Liu, Zhen Wu, Tingting Shao, Wanzhen Zheng and Jing Huang *
Department of Integrated Traditional Chinese & Western Medicine Oncology, Hangzhou Cancer Hospital, Hangzhou, China

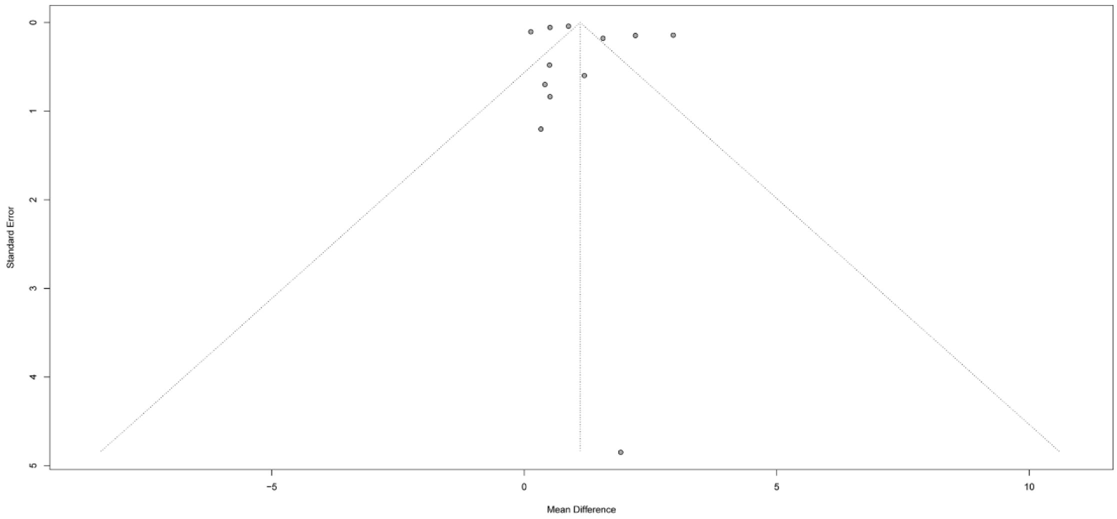

Figure S1. Funnel Plots for detecting publication bias in the 12 included studies of the meta-analysis.

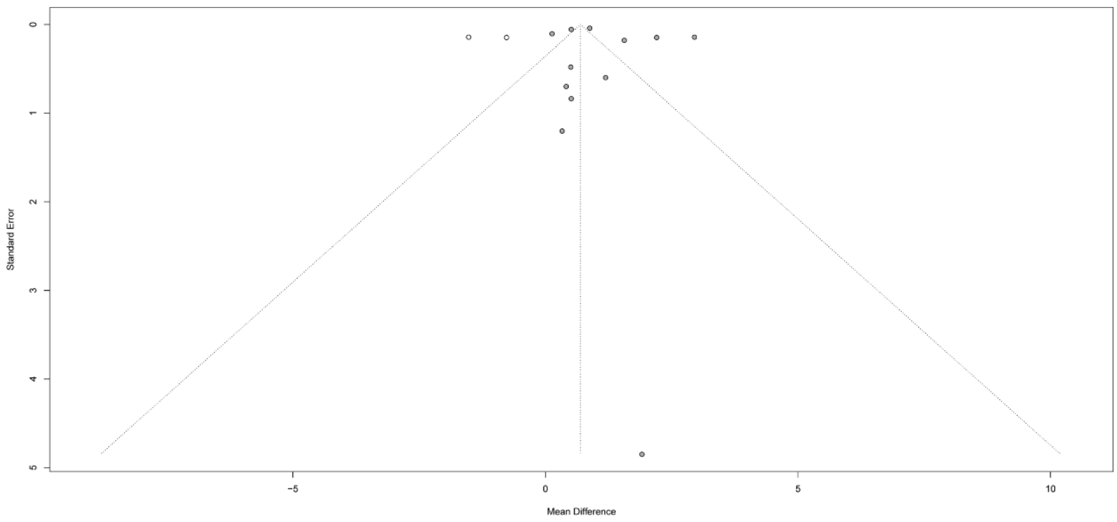

Figure S2. Funnel plots of publication bias analysis adjusted by the trim-and-fill method.

Study	Mean Difference	MD	95%−CI	P−value	Tau2	Tau	I2
Omitting Miyazaki 2021		1.13	[0.48; 1.79]	< 0.01	0.8986	0.9479	97%
Omitting Meng 2021		1.05	[0.40; 1.70]	< 0.01	0.8860	0.9413	97%
Omitting Tan 2021		1.24	[0.63; 1.84]	< 0.01	0.7438	0.8624	97%
Omitting Jantharapattana 2020		1.14	[0.53; 1.75]	< 0.01	0.8292	0.9106	97%
Omitting Yang 2020		0.98	[0.37; 1.59]	< 0.01	0.7556	0.8693	97%
Omitting Zhu 2019		0.90	[0.42; 1.39]	< 0.01	0.4426	0.6653	94%
Omitting Ritch 2019		1.10	[0.51; 1.70]	< 0.01	0.8082	0.8990	97%
Omitting Aoyama 2019		1.16	[0.53; 1.79]	< 0.01	0.8478	0.9207	97%
Omitting Feijó 2019		1.18	[0.54; 1.82]	< 0.01	0.8450	0.9193	97%
Omitting Ida 2017		1.14	[0.52; 1.76]	< 0.01	0.8437	0.9185	97%
Omitting Hatao 2017		1.16	[0.54; 1.78]	< 0.01	0.8385	0.9157	97%
Omitting Imamura 2016		1.10	[0.46; 1.73]	< 0.01	0.8808	0.9385	97%
**Random effects model**		**1.11**	**[0.52; 1.70]**	**< 0.01**	**0.8043**	**0.8969**	**97%**

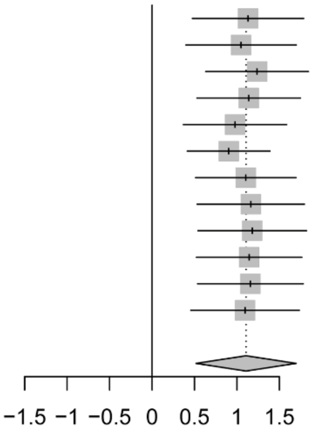

Figure S3. Forest plots depicting sensitivity analysis to evaluate the impact of study exclusion on meta-analysis outcomes.
